# “I only knew how to search Google”: students’ reflections on a four-year information literacy curriculum

**DOI:** 10.29173/jchla29808

**Published:** 2025-08-01

**Authors:** Stephanie Sanger, Denise A. Smith

**Affiliations:** 1 MLIS, Clinical Outreach Librarian, Health Sciences Library, McMaster University, Hamilton, ON; 2 MLIS, Head, Research Lifecycle, Brock University Library, St Catharines, ON

## Abstract

**Introduction:**

For undergraduate general health sciences education, there is insufficient high-quality evidence that speaks to the benefits and challenges of an integrated and scaffolded information literacy (IL) curriculum when compared to more traditional modes of delivery. Calls for scaffolded and curriculum-integrated programs are on the rise.

**Objectives:**

This study aims to understand students’ perceived confidence and skill levels when engaging with or searching for health information, after four years of IL training.

**Methods:**

A mixed-methods survey was used to learn whether or how students’ confidence in their IL skills was impacted by an IL curriculum integrated into the Bachelor of Health Sciences (BHSc) program at McMaster University. Quantitative survey questions were analysed with descriptive statistics. Qualitative survey questions underwent three cycles of coding to identify themes in participant responses to open-ended questions.

**Results:**

Of 194 fourth-year students, 49 participated in the survey, a response rate of 25%. 79% of participants recalled feeling “unconfident” or “not so confident” in finding information in their first year of study. By their fourth year, all participants indicated they felt “somewhat confident” (53%) or “very confident” (47%). 93% of participants indicated their confidence in searching for information was positively impacted by the mandatory IL curriculum. 90% of participants believed they would use these IL skills after graduation.

**Conclusion:**

A mandatory curriculum-integrated IL program in undergraduate health sciences positively impacted students’ confidence in searching for and finding information. This study indicates the potential benefits of integrating IL instruction into program-wide curricula for undergraduate students.

## Introduction

### Background

In the Spring of 2023, the first cohort to complete a four-year, curriculum-integrated information literacy (IL) program graduated from the Bachelor of Health Sciences (BHSc) program at McMaster University. The program description for this curriculum was published in 2023, which provides a complete description of the multi-year embedded IL curriculum that was scaffolded to build rudimentary IL skills and introduce key conversations around information production and dissemination [1].

The BHSc program is highly competitive for first-year entry. For example, in 2024, the program received a record number of approximately 7000 applications for the 2024-25 academic year and the program accepted approximately 250 students. For the same admissions calendar, 14 second-year students were accepted into the BHSc program as transfer students. Students who enter the BHSc program at McMaster University typically graduate from secondary education at the top of their class. They are high-achieving and disciplined students who trend towards being highly self-motivated.

The Health Sciences Library and the BHSc Program at McMaster University have worked together since 2017 to develop a for-credit, scaffolded IL program that is embedded into a mandatory four-year course called Praxis Pathways, which is a holistic learning course crafted around four “threads”. One of these threads was called Information Literacy and was designed by librarians. Praxis Pathways is the first undergraduate course in the Faculty of Health Sciences to embed an IL curriculum. When the first cohort of BHSc students who completed the entire Praxis Pathways curriculum were situated to graduate, the authors developed a research study to learn about the potential impact this curriculum may have had on these students’ confidence in their IL skills. The protocol for this study was reviewed by the Hamilton Integrated Research Ethics Board (HiREB) under project ID 15335.

### Literature review

Johnston and Webber (2003) define information literacy as:

The adoption of appropriate information behaviour to obtain, through whatever channel or medium, information well fitted to information needs, together with critical awareness of the importance of wise and ethical use of information in society [2].

The integration of IL within postsecondary academic curricula is a longstanding and ongoing discussion in librarianship and library practice literature [3–6]. The introduction of the “ACRL Framework for Information Literacy for Higher Education” (2015) provides academic librarians with six frames upon which IL curricula can be built and that librarians can use when building instructional sessions for students. Around the same time the new “Framework” was released, metaliteracy emerged [7], as a conceptual model that examines one’s ability to know what one doesn’t know. In other words, information literacy is just one form of literacy nested within a broader ability to understand, articulate, and respond to a need. The “Framework”, and metaliterary as a grounding concept, can frame IL education, and has positioned IL as a central function of university libraries. However, there are unique considerations for health sciences libraries and their approach to IL education, which are influenced by the overall learning objectives and key competencies for graduates of health sciences programs [8].

Undergraduate health sciences education calls on learning objectives such as those found in medical professional education [9,10], namely evidence-based practice [11,12] and problem-based learning [13,14]. However, among the literature about information literacy within health sciences education, undergraduate nursing education is strongly represented, with limited attention to undergraduate health sciences as an educational program [1]. Amongst the limited body of literature focusing on health sciences and IL [15–19], one study found that to have a long-term impact on undergraduate health sciences education, it is best to teach IL skills gradually throughout the entirety of an educational program. The study argues that this approach helps students become lifelong learners who can use these skills in different situations [20]. Embedding IL skills throughout an entire academic program is well supported, and there is evidence that it has been applied in a broad range of academic disciplines within postsecondary education [21]. In nursing education, for example, this has taken the form of scaffolding evidence-based practice instruction, which inherently includes IL development for nursing students [22].

### Objectives

This study sought to gain insight into the impact of a curriculum-integrated IL program on students’ perceived confidence in their IL skills. Namely, students’ perceived confidence in searching for information in the context of their academic studies. The study was guided by the following research questions:

1. Did the IL program influence BHSc students' confidence in their IL skills?
2. How did BHSc students perceive their IL in the first year, compared to after their fourth year?

The authors’ primary goal was to develop an understanding of students’ perceived confidence when engaging with or searching for health information after four years of formal IL instruction. The authors’ secondary goal was to gain insight into the students’ perceived IL skill levels when engaging with or searching for health information after the same period. These two goals support the authors’ understanding of the impact of the IL curriculum on students, particularly to gain insight into how it might impact their confidence in information searching and retrieval as they move beyond undergraduate education.

## Methods

The authors created a mixed methods survey (Appendix - Survey instrument) that employed multiple choice questions to collect anonymous quantitative data and open-ended questions to collect anonymous qualitative data. The survey asked participants to express their interest in participating in a focus group, but the authors did not receive enough interest from participants to move forward with focus groups.

In May 2023, an invitation to participate in a survey was distributed via email and among online learning spaces to all fourth-year BHSc students (n=194). Print advertisements were posted in primary learning spaces for the program. To be eligible to participate students were required to self-identify as a Level IV BHSc student. Only participants who answered “yes” to this self-verification question gained access to the survey. Students who answered “no” were not eligible to complete the survey and were not granted access to any survey questions beyond self-verification. The survey was administered online with Microsoft Forms and was open for six weeks between May 2023 and June 2023. Students who completed the survey were invited to enter their name into a separate form to be entered into a draw to win one of three available sets of AirPods.

Survey responses were divided into qualitative questions (n=3) and quantitative questions (n=8) for analysis. Quantitative questions were analysed in a spreadsheet to produce descriptive statistics about the respondent answers to quantitative questions. No inferential statistical tests were performed as part of this study. Qualitative questions underwent thematic analysis. Each question underwent three cycles of open coding, primarily creating “in vivo” codes to represent the voices of study participants. Descriptive and concept codes were also employed when deemed appropriate. All codes were created by consensus between both authors. The third cycle of coding resulted in the establishment of a single overarching theme in student responses to each of the three qualitative questions.

## Results

25% (n=49) of eligible students (n=194) responded to the authors invitation to participate in the survey. All 49 respondents met the study’s inclusion criteria and completed the survey, producing a convenience sample of the target population. The results can be divided into two categories based on the above stated research questions: students’ perceived confidence in their IL skills and the impact of Praxis Pathways; and student perceptions of IL.

### Impact of Praxis Pathways IL curriculum on IL confidence

When asked to rate their confidence in approaching library research when they were a first-year student, 80% (n=39) of respondents indicated they recalled feeling “unconfident” or “not so confident” and only 6% (n=3) recalled feeling “somewhat confident”. When asked to rate their confidence after completing their fourth year of study, 100% (n=49) of students indicated they felt either “somewhat confident” or “very confident”.

These responses indicate a change in students’ perceived confidence in their IL skills between entering the BHSc program in their first year of study and upon departure from the program after their fourth year of study.

When asked to rate the impact the Praxis Pathways curriculum had on their confidence in searching for information, 51% (n=25) of respondents indicated the curriculum was “somewhat impactful” and 43% (n=21) indicated it was “very impactful”.

Students were also asked to select, from a list, all other resources available to them at the university that they perceive as having contributed to their improved IL confidence. Professors or course facilitators, other courses, and peers or friends were each selected by more than half of all respondents, with librarians selected by 31% of respondents (n=15). Other Praxis Pathways threads, general library workshops and visiting the library information desk were selected by 24% (n=12) or fewer respondents.

### Student perceptions of IL

Survey participants were asked whether they thought they needed to learn how to find information when they entered their first year of the BHSc Program. 59% (n=29) responded “yes” while 41% (n=20) responded “no”. Respondents who answered “yes” were branched to an open-ended question that asked what IL skills they thought they needed to learn when they entered the first year of their program. Respondents who answered “no” were branched to an open-ended question that asked why they believed they did not need to learn IL skills.

Among those who answered “yes” to whether they thought they needed to learn how to find information when they entered their first year, survey respondents generally reported that they were aware there were skills or knowledge they did not have (“I knew there were skills I didn’t have”) but the degree to which they knew what they did not know, their metaliteracy [21], varied among respondents.

This metaliteracy ranged from a vague awareness that “there were skills [the student] didn’t have or know” (Participant #05), to a more specific awareness of a knowledge gap such as, “how to find reliable sources to apply to research projects” (Participant #21). The survey responses suggest that students can arrive at university with some awareness that they will need to be able to search for information, but their understanding of this knowledge gap falls on a spectrum. The authors named this theme “I knew there were skills I didn’t have/know”, which suggests that this group of survey participants entered university aware of an existing gap in their knowledge around how to conduct searches and find information relevant to the research requirements of their program or projects.

Among survey participants who answered “no”, they did not believe they needed to know how to find information when they entered their first year of the BHSc Program, another broad theme emerged. These students expressed a general lack of preparation or awareness of the requirements of academic research, particularly related to literature searching and the library-related skills required to support academic work. The authors named this theme “I didn’t realize”.

Similar to “I knew there were skills I didn’t have/know”, the findings amongst this group of respondents also indicate a knowledge gap. However, this knowledge gap is perhaps more insidious than the above finding because these students “didn’t realize” they would need to know how to search for information. The phrase “I didn’t realize” is woven into several responses. Students also called on their assumptions about university, their past experiences such as high school, and their experience searching Google. Participant #51 expressed this overall theme well in their statement:

I was completely unaware of what the ‘literature’ or databases even were, and that there was a whole art to searching beyond just the simple Google searches I did in high school.

Based on the reported changes in participants' perceived confidence in their IL skills and their perceptions of whether or not they needed to learn how to search for information, it can be inferred that Thread 4 Praxis Pathways provided BHSc students with instruction and skills they did not have upon entering the program and this instruction improved their confidence when finding and evaluating information.

Finally, when asked the open-ended question, “what skills or knowledge have you learned in Thread 4 Praxis Pathways that you think will be useful after you graduate” participant responses fell within one of two emergent themes: information searching and retrieval (and its various components), or they referred to a specific upper year module. Most of the upper year learning modules explore information searching and retrieval, while some incorporate additional critical thinking about health information creation and dissemination [1]. “Databases” was the most commonly provided response (n=35), followed by “information searching” (n=16) and “using the correct search terms” (n=9). A complete list of response themes is available in Table 6. The frequent occurrence of “databases” and “information searching” as skills students expect to use after graduation provide richer insight into the impact of this curriculum on students’ IL skills and their confidence in finding information.

**Table 1:** Perceptions of IL in year 1 and year 4

	Year 1 (n=)	Year 1 (%)	Year 4 (n=)	Year 4 (%)
Unconfident	13	27%	0	0%
Not so confident	26	53%	0	0%
Neutral	7	14%	0	0%
Somewhat confident	3	6%	26	53%
Very confident	0	0%	23	47%
TOTAL	49	100%	49	100%

**Table 2:** Impact of Praxis Pathways curriculum on IL confidence

	n=	%
No impact. I did not learn anything new.	0	0%
Not very impactful	1	2%
Neutral	2	4%
Somewhat impactful	25	51%
Very impactful. I learned a lot that built my confidence.	21	43%
TOTAL	49	100%

**Table 3:** Other sources of improved IL confidence

	n=	%
Professors or course facilitators	38	78%
Other courses	32	65%
Peers or friends	28	57%
Librarian consultations	15	31%
Other Praxis Pathways threads	12	24%
General Library workshops	9	18%
Visiting the Library information desk	8	16%

**Table 4:** Theme: “I knew there were skills I didn’t have/know”

Subtheme	Occurrences (n=)	Examples	Participant #
Forfuture research projects	3	“How to conduct literature searches in hopes of becoming involved with research projects”	18
How to find	35	“Not just searching on Google”	12
		“Where to look, what information is credible”	53
		“Finding specific articles that were relevant and current”	20
How to conduct literature searches	15	“How to use databases etc... I only had experience with Google and Google Scholar so I knew I needed to learn more but didn’t really know what else was out there”	13
		“I knew there were formal search strategies and skills I didn’t have/know”	7
		“I only knew how to search Google for scientific information and had no idea how to use databases”	46
		“How to use the right search terms”	16

**Table 5:** “I didn’t realize”

Subtheme	Occurrences (n=)	Examples	Participant #
“I didn’t realize” -assumptions about university	23	“I didn’t realize I would be involved with searching so much literature throughout my course. I didn’t really know that research was such a big part of my work”	09
		“I thought most material would be provided to us and the program would be less research-intensive”	42
		“University was just never portrayed in such a way, and I guess I never knew how big of a part search for information plays”	11
Past experience	6	“I thought I already knew how to search the internet on my own”	29
		“I wasn’t formally introduced to research until university and so finding scholarly information didn’t happen to be a big factor of my academic journey”	33
“I thought Google would be sufficient”	10	“Before Praxis Pathways I was only using Google Scholar to find academic sources and I didn’t realize there was more to it”	17
		“In most contexts simple Google searches had allowed me to find the information I needed”	25
		“Based off past experience, I don’t think I even knew about the world of finding information -I naively thought that a google search would suffice”	34

**Table 6:** Information searching and retrieval skills

Subtheme	Occurrences
Databases	35
“Information searching”	16
“Using the correct search terms”	9
“How to use Boolean operators”	8
“Reliable sources”	8
“How to add limits and narrow my search”	4
“Critical thinking”	2
Reference management	1

## Discussion

### Limitations

Limitations of this study can be associated with the participation rates, study type, and the inability to gather richer data using focus groups. The response rate for this study did not represent the entire cohort of students, which yielded a response rate of 25% (n=49) from a possible 194 students.

Additionally, retrospective study types come with the chance that participants could be susceptible to hindsight bias. Participants’ recollections are at risk of being recalled through the lens of what the students know now. Future longitudinal studies into the impact of this, or similar curricula may benefit from an initial survey of first-year students as they enter the program, followed by the same survey as they exit four years later.

Finally, the original methodology proposed for this study, which intended to use focus groups to gather data, gathered little interest from survey participants. Had enough students expressed interest to run the focus groups, the authors might have received richer insight into the students’ perceived confidence levels when searching for information, changes in their confidence over time, and their perception of the value of the Praxis Pathways curriculum.

### Conclusion

This study explored the impact of an IL curriculum offered over four years of an undergraduate health sciences program at McMaster University. It adds to existing literature and research on the impact of IL instruction in health education by providing insight into the impact of a scaffolded curricula in undergraduate health sciences. Given that the literature on IL in health education has a strong focus of IL instruction in nursing programs, this study provides an additional context for understanding the value of librarian-led IL training in post-secondary health sciences programs.

The authors reported that 79% of respondents described lacking confidence in their information skills as first-year students. Compared with the 100% of respondents who indicated feeling somewhat or very confident after completing their fourth year. This study suggests the potential for scaffolded (where each year of instructional content builds upon the last) and curriculum-integrated IL education to offer a positive outcome for undergraduate students. Moreover, this increased confidence in IL skills among graduates of the BHSc program has implications as they move into professional education (e.g. medical school), graduate school, or professional careers in the health sector. For example, confidence in searching for health information could potentially influence graduates' performance in their postgraduate academic or professional careers as they engage in real-world scenarios that require a comfort with navigating, searching, and retrieving health information.

While the results of this study indicate the impact of this program’s offerings on the students who participated in the survey, its observations are worthy of future investigation. As reported, 41% of survey respondents shared that they did not anticipate a need to learn IL skills when they first entered university. Although 59% anticipated they would need these skills, these respondents also acknowledged they had a knowledge gap in this regard. Therefore, all participants indicated that they were unprepared for information searching when entering university, whether they realized it or not.

Whether students entered university aware they lacked sufficient IL skills, or were unaware of their knowledge gap, a single overarching theme emerged that suggests to the authors that incoming post-secondary students may be insufficiently prepared for post-secondary study when it comes to searching for, finding, retrieving and engaging with information in higher education. Furthermore, these skills, once acquired, have the potential to be carried forward as students move forward in their collegiate or professional careers.

Given the knowledge that BHSc students are highly attuned to what is required in academia and are not adequately prepared for academic research, the authors ask: what does this suggest for postsecondary students who enrol in less competitive programs? What can high schools and universities do to prepare students for undergraduate-level research? A larger-scale study on the IL of students entering their first year of study is recommended. More research is needed to understand how secondary institutions are preparing their students for university. In the meantime, academic libraries in post-secondary institutions might consider how they could account for this knowledge gap among students entering university in their programming for first-year students, and how to incrementally develop these necessary skills.

## Data Availability

Raw anonymized data collected for this study is available through the Brock University Dataverse (Borealis) [23].

## Appendix - Survey instrument

**Table:**

1	M/C	Are you currently enrolled in BHSc Level IV? Yes →proceed to question 2 No →end survey
2	Lykert	Think about your confidence level searching for information when you started the BHSc program in your first year. Please rate your confidence in approaching library research when you were a first-year student. 1 = No confidence, 3 = Somewhat confident, 5 = Very confident
3	M/C	When you first started university, did you think you needed to learn how to find information? Yes → Proceed to 4 No → Proceed to 5
4	Short Ans.	What did you think you needed to learn?
5	Short Ans.	Why did you believe this?
6	Lykert	Think about your confidence level searching for information now that you are nearing completion of the BHSc program. Please rate your confidence in approaching library research. 1 = No confidence, 3 = Somewhat confident, 5 = Very confident
7	Lykert	Think about the Information Literacy thread (Thread 4) in Praxis Pathways. Please rate its impact on your confidence in searching for information. 1 = No impact. I did not learn anything new, 3 = Some impact. I learned some new things that built my confidence. 5 = Very impactful. I learned many new things that built my confidence.
8	Select all	Beyond Praxis Pathways Thread 4, what other sources enhanced your confidence in searching and retrieving information? Please select all that apply. Friends or peers Professors or course facilitators Other Praxis Pathways Threads Other courses General library workshops Visiting the library help desk Librarian consultations
9	Select all	Please select which modules you completed in 3X00 and 4XP3.
10	Select all	Of the modules you **completed**for Thread 4 from BHSc Level I to IV, which do you suspect will be most useful after you graduate from BHSc?
11	Select all	Of the modules you **completed**for Thread 4 from BHSc Level I to IV, which did you suspect will be least useful after you graduate from BHSc?
12	MC	Reflect on what you have learned from Thread 4 Praxis Pathways. Do you think you will use what you have learned after you graduate? Yes → Proceed to question 13 No → Proceed to question 14
13	Short Ans.	What skills or knowledge have you learned in Thread 4 Praxis Pathways that you think will be useful after you graduate?
14	Short Ans.	Do you have any additional comments or feedback for Thread 4 Praxis Pathways?If yes, please share below. If no, please type “N/A” in the response field.
15	Long Ans.	Feedback
